# Position Effects of Metal Nanoparticles on the Performance of Perovskite Light-Emitting Diodes

**DOI:** 10.3390/nano11040993

**Published:** 2021-04-13

**Authors:** Chen-Min Yang, Fang-Chung Chen

**Affiliations:** 1 Department of Photonics, College of Electrical and Computer Engineering, National Chiao Tung University, Hsinchu 30010, Taiwan; dennis51025@gmail.com; 2 Center for Emergent Functional Matter Science, National Chiao Tung University, Hsinchu 30010, Taiwan; 3 Department of Photonics, College of Electrical and Computer Engineering, National Yang Ming Chiao Tung University, Hsinchu 30010, Taiwan

**Keywords:** plasmonics, nanoparticle, perovskite, photoluminescence, electroluminescence

## Abstract

Metal nanoparticles have been widely used for improving the efficiencies of many optoelectronic devices. Herein, position effects of gold nanoparticles (Au NPs) on the performance of perovskite light-emitting diodes (PeLEDs) are investigated. Amphiphilic Au NPs are synthesized so that they can be incorporated into different layers of the PeLEDs to enhance device efficiencies. The photoluminescent (PL) studies indicate apparent position effects; the strongest PL intensity occurs when the NPs are directly blended with the light-emitting perovskite layer. In contrast, the PeLEDs exhibit the highest luminance efficiency while the Au NPs are placed in the hole-transporting layer. The direct blending of the NPs in the perovskite layer might affect the electrical properties, resulting in inferior device performance. The results reported herein can help to understand the enhancing mechanism of the PeLEDs and may also lead to even better efficiencies in the near future.

## 1. Introduction

Organic–inorganic hybrid perovskite materials are receiving increasing attention for many optoelectronic applications because of their advantageous properties, including high absorption coefficients, high carrier mobilities, solution processability and low material costs [1,2,3,4]. The power conversion efficiencies of perovskite solar cells (PSCs) have been improved rapidly, from the first reported value of 3.8% to the current record of 25.5% [5,6]. For many other device applications, such as light-emitting devices (LEDs) [7,8,9], lasers [10,11], and photodetectors [12], perovskite materials have also been widely adopted to construct next-generation optoelectronic devices. In particular, perovskite materials possess high photoluminescence quantum yield (PLQY) and narrow emission over the entire visible spectrum; their high color purities make them promising candidates for future displays and lighting sources [8].

The device efficiency of the earliest perovskite light-emitting diodes (PeLEDs) was very low, and the electroluminescence (EL) could only be observed at liquid nitrogen temperatures [13]. Currently, external quantum efficiencies (EQEs) over 20% have been reported [14]. However, their EQEs are still lower than those of inorganic LEDs and organic light-emitting diodes. Therefore, further improvements in the device efficiencies are still required if PeLEDs can compete with other LED technologies. Among the proposed strategies for improving the device EQEs of PeLEDs, the use of plasmonic nanostructures has been considered a promising method [8]. For example, Ag nanorods were incorporated into the hole-transporting layers of CsPbBr_3_-based PeLEDs to improve the device efficiencies in 2017 [15]. The EQE was improved up to 43.3%, and the authors attributed the device improvement to the increased radiative recombination rate induced by the plasmonic field of the nanorods. Meanwhile, Chen et al. blended Au nanoparticles (NPs) into the hole buffer layer, poly(3,4-ethylenedioxythiophene)-poly(styrenesulfonate) (PEDOT:PSS), and found significant improvement in the device efficiencies [16]. The authors optimized the CsPbBr_3_-based PeLEDs through quenching control, and 86% enhancement in the maximum EQE was achieved. On the other hand, the device-enhancing mechanism of these plasmonic-enhanced PeLEDs is still not fully understood. Furthermore, there are only a few studies about plasmonic-enhanced PeLEDs. Therefore, in this work, we prepared amphiphilic Au NPs that can be dispersed well in both aqueous and organic solvents [17]. Their amphiphilicity allowed us to incorporate them into different layers of the PeLEDs, thereby facilitating the investigation of the position-dependent effects on the performance of PeLEDs. The experimental results reveal the device performance is enhanced if the metal NPs are incorporated closer to light-emitting layers (LELs). However, direct blending of the NPs into the perovskite layers might lead to morphology and/or changes in electrical properties, thereby degrading the device efficiency. As a result, the best location for the Au NPs is found to be in the hole-transport layer (HTL), which was closer to the LELs. Because of such position dependence, we deduce that the near field nature of the plasmonic field, which theoretically decays exponentially with the distance from the metal surface, should be responsible for the device improvements. The results of this work should improve the understanding of the mechanism behind the device enhancement of the plasmonic PeLEDs.

## 2. Materials and Methods

### 2.1. Materials

All the chemicals were used as received. Hydrogen tetrachloroaurate(iii) trihydrate (HAuCl_4_·3H_2_O) was purchased from Alfa Aesar. 9,9-Bis[4-[(4-ethenylphenyl)methoxy]phenyl]-N2,N7-di-1-naphthalenyl-N2,N7-diphenyl-9H-fluorene-2,7-diamine (VB-FNPD), poly[bis(4-phenyl)(2,4,6-trimethylphenyl)amine] (PTAA) and 2,2′,2″-(1,3,5-Benzinetriyl)-tris(1-phenyl-1-H-benzimidazole) (TPBi) were obtained from Lumitec. Other chemicals were purchased from Sigma-Aldrich. The amphiphilic Au NPs were synthesized following our previous method [17]. In short, an aqueous solution of HAuCl_4_ was mixed with a solution containing the as-prepared PEGylated graphene oxides (PEG−GO). Then, sodium citrate was added to the solution to reduce the Au ions. The mixture was heated to 80 °C and stirred for 4 h. The suspension was centrifuged (6000 rpm), and the residue was washed twice with deionized water to remove any unreacted starting material. Finally, the amphiphilic Au NPs were dried through lyophilization.

### 2.2. Device Fabrication and Characterization

**Figure 1:**
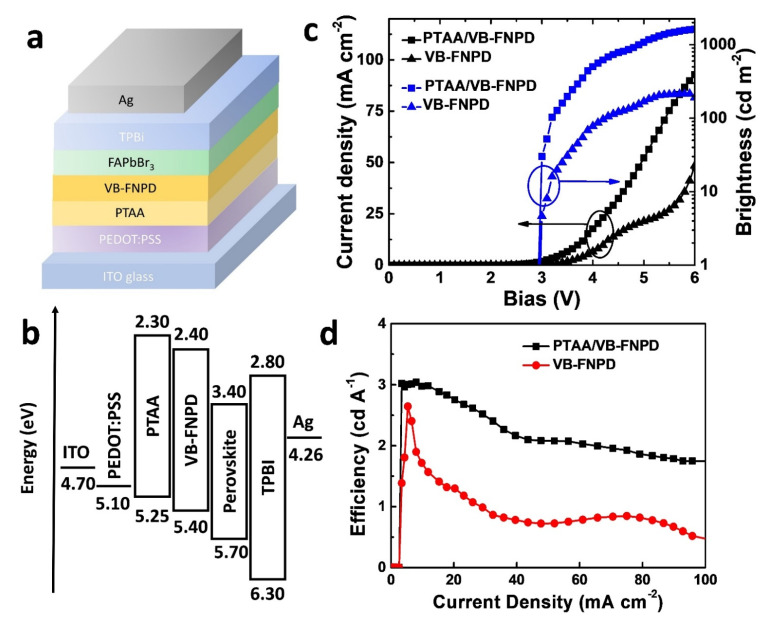
Characterizations of the perovskite light-emitting diodes (PeLEDs) in this study: (**a**) the device structure; (**b**) energy levels of the materials and work functions of electrodes in this work; (**c**) the electrical properties of the PeLEDs prepared with single and double hole-transport layers (HTLs); (**d**) the device efficiencies of the PeLEDs as a function of the current density.

The PeLEDs were fabricated on indium–tin–oxide (ITO)-coated glass substrates; Figure 1a displays the device structure. After cleaning, the substrates were dried overnight at 100 °C. Right before device fabrication, the substrates were exposed to UV ozone treatment for 15 min. An anodic buffer layer of PEDOT:PSS was deposited through spin-coating on to the ITO substrates, followed by thermal annealing at 150 °C for 15 min. As shown in the diagram of the energy levels (Figure 1b), the active perovskite layer exhibits a low-lying valence band edge. Therefore, we used a two hole-injection/transport layer to improve the hole injection efficiency. PTAA was dissolved in a solvent mixture, consisting of toluene and dimethylformamide (DMF) (2:1, v/v); the concentration was 2.0 wt %. The PTAA solution was spin-coated on the PEDOT:PSS layers at 2500 rpm for 60 sec and the resulting sample was annealed at 100 °C for 30 min. The second HTL of VB-FNPD was spin-coated from DMF (2.0 wt %) at 2000 rpm for 60 sec and was subject to pre-annealing at 100 °C for 30 min. Furthermore, the VB-FNPD layer was cross-linked by annealing at 230 °C for 90 min. Note that the addition of DMF in the solvent system dissolving PTAA could improve the wetting of VB-FNPD on the PTAA layer. Then, we fabricated FAPbBr_3_ thin films on the HTLs as the LEL of the PeLEDs. The LEL was prepared using a two-step preparation method. PbBr_2_ and formamidinium bromide (FABr) were firstly dissolved in DMF (0.75 M) and isopropanol (IPA, 6.0 mg mL^−1^), respectively; both solutions were stirred for more than 24 h. To prepare the perovskite layers, the PbBr_2_ solution was spin-coated onto the surface of the VB-FNPD layers at 7000 rpm; the sample was heated at 75 °C for 10 min. Then, the FABr solution was over-coated on the previous PbBr_2_ layers at 7000 rmps and the resulting samples were annealed at 75 °C for 3 min. As for the electron-transport layer, TPBi was dissolved in chlorobenzene (CB) at a concentration of 2.0 wt %. The solution was spin-coated on the previous perovskite layers at 2000 rpm for 60 sec; they were annealed at 75 °C for 10 min. Finally, the device was completed by depositing a Ag layer (80 nm) through thermal evaporation under a vacuum of 6 × 10^−6^ torr. The active area of the fabricated device was 10 mm^2^. For the devices containing Au NPs, various amounts of NPs were dispersed into the different solutions as described previously. Other fabrication procedures for devices containing Au NPs were similar.

All the PeLEDs were encapsulated in the glove box using cover glasses and sealed with UV-cured epoxy prior to characterization. The device characterization was performed in air. The device electrical characteristics of the PeLEDs were measured using a Keithley 2400 source-measure unit and a PR655 SpectraScan^®^ colorimeter (Photo Research, Chatsworth, CA, USA). Absorption spectra were measured using a UV−Vis−NIR spectrometer (PerkinElmer Lambda 950, PerkinElmer, Waltham, MA, USA). Time-resolved photoluminescence (TRPL) measurements were performed by using a PicoQuant system consisting of a 375 nm laser equipped with a PDL 200-B picosecond pulsed diode laser driver, a time-correlated single photon counting system, a spectrometer (iRH-550, Horiba, Kyoto, Japan), and a fluorescence microscope.

## 3. Results and Discussion

Figure 1a displays the device structure used in this work. The PeLEDs were fabricated on indium–tin–oxide (ITO)-coated glass substrates [18,19]. Figure 1b revealed the energy levels of the materials and work functions of the electrodes. Because of the large hole-injection barrier, we previously used VB-FNPD as the single HTL in the PeLEDs to improve the hole-injection efficiency [18]. However, from the energy-level diagram (Figure 1b), we still could notice a large barrier existing between the work function of PEDOT:PSS and the highest occupied molecular orbital (HOMO) of VB-FNPD. In order to further improve the hole injection efficiency, we inserted the other HTL, poly[bis(4-phenyl)(2,4,6-trimethylphenyl)amine] (PTAA), between the PEDOT:PSS and VB-FNPD layers. In principle, such a ladder-type energy structure can facilitate hole injection in the PeLEDs. Figure 1c shows the current density−brightness−voltage (*J*–*B*–*V*) curves of the PeLEDs prepared with single and double HTLs. From the *J*–*V* curves, we observed that the current density of the device prepared with double HTLs apparently increased after the bias was larger than 2.5 V. On the other hand, from the results of current density, the device prepared with a single HTL turned on more slowly at 2.9 V, indicating that the addition of the PTAA layer could indeed promote the hole injection into the PeLEDs. However, if we defined the light turn-on voltage (*V*_LT_) as the one at which the brightness was 0.1 cd m^−2^, the *V*_LT_ values of the PeLED prepared with single and double HTLs were 3.0 and 2.9 V, respectively [17]. More importantly, the PeLEDs prepared with the double HTLs exhibited higher efficiency. As revealed in Figure 1d, the highest luminance efficiency (*L*_E_) of the device prepared with a single HTL layer was 2.6 cd A^−1^; the efficiency decayed rapidly with the increasing current density and was decreased to 1.0 cd A^−1^ at about 30 mA cm^−2^. For the double HTL device, the highest *L*_E_ was up to about 3.1 cd A^−1^, presumably due to the better charge balance. The efficiency also decreased more slowly than the device prepared with a single HTL. Therefore, we employed the structure with double HTLs for fabricating the standard PeLEDs in the following works.

**Figure 2:**
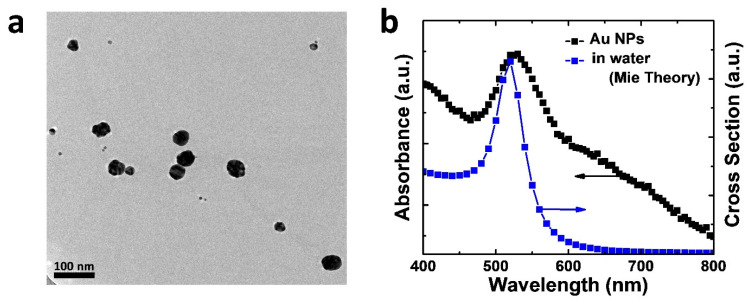
Characterizations of the Au nanoparticles (NPs): (**a**) transmission electron microscopy (TEM) image of the NPs; (**b**) the absorption spectrum of the Au NPs dispersed in water. The simulated cross-section of the Au NPs using Mie theory is also displayed for comparison.

In order to enhance the performance of PeLEDs, we synthesized amphiphilic Au NPs [17]. Figure 2a shows the transmission electron microscopy (TEM) image of the as-prepared Au NPs. The average diameter was about 30 nm. We also observed that the density of the Au NPs was slightly higher at the edges and at the wrinkles on the planes of graphene oxides (GO); we suspect that the higher numbers of defect sites (e.g., carboxyl groups) provided greater amounts of net negative charge for immobilizing the Au ions. These properties were consistent with our previous results [17]. Figure 2b shows the absorption spectrum of the Au NPs in water. We observed a peak at 545 nm, which is the typical location of the localized surface plasmon resonance (LSPR) for Au NPs. Furthermore, we simulated the extinction cross-sections of the Au NPs using Mie theory [20,21]; the resulting spectrum is also displayed in Figure 2b. Assuming the diameter of the Au NPs was 30 nm, the simulated peak of the plasmonic band was located at 520 nm when they were dispersed in water (refractive index *n* = 1.33). The peak position was close to the experimental result, suggesting the formation of Au NPs.

**Figure 3:**
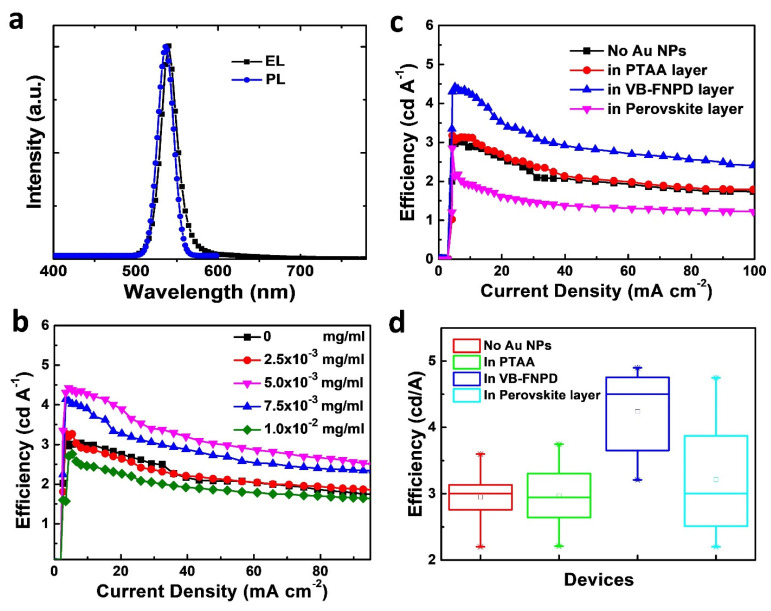
(**a**) Electroluminescent (EL) and PL spectra of the PeLEDs in this study. (**b**) Luminance efficiency-current density (*L*_E_-*J*) characteristics of the PeLEDs prepared with different amounts of Au NPs in the 9,9-Bis[4-[(4-ethenylphenyl)methoxy]phenyl]-N2,N7-di-1-naphthalenyl-N2,N7-diphenyl-9H-fluorene-2,7-diamine (VB-FNPD) layers. (**c**) *L*_E_-*J* curves of the PeLEDs for the devices containing Au NPs at different device locations. (**d**) Box plots of the device efficiency for the PeLEDs prepared under various conditions.

To improve the device efficiencies, we firstly blended the Au NPs in the VB-FNPD layers. Figure 3a shows the typical electroluminescent (EL) spectrum of the PeLEDs. The EL spectrum is almost identical to the photoluminescent (PL) spectrum, suggesting the same electronic state was excited. The EL spectrum was actually unchanged for all the devices we reported in this work. Figure 3b illustrates the efficiencies of the PeLEDs prepared with various amounts of Au NPs. While a very small amount of Au NPs were added (2.5 × 10^−3^ mg mL^−1^), the efficiency was almost unaffected. At the optimal concentration (5.0 × 10^−3^ mg mL^−1^), the device efficiency was improved and an efficiency of 4.4 cd A^−1^ has been achieved (Figure 3b); the efficiency was improved averagely by up to 42%. The efficiency of the best device could reach higher than 4.9 cd A^−1^. If we further increased the concentration, the device efficiency started to decrease. The peak of the EL spectrum was located at 548 nm, which was very close to the position of LSPR of the Au NPs. Therefore, we deduced that the strong coupling between the excitons and the LSPR improved the performance of PeLEDs.

If we consider the multiple-layer structure of the PeLEDs, there are other possible positions where we can place the Au NPs within the devices. Because the plasmonic electrical field decays rapidly with the distance away from the surface of the metal NPs, the spatial location of plasmonic NPs critically affects the efficiency of a device [17]. Ideally, if we blended the Au NPs directly into the perovskite LELs, the device should exhibit the best performance due to the direct contact between the NPs and the light-emitting species [22]. Unfortunately, as shown in Figure 3c, the device efficiency was decreased after we blended the Au NP into the LELs. Although the highest efficiency was almost the same as that of the standard device, the *L*_E_ values became lower while the current density was larger. Figure 3d illustrates the statistical data of the device efficiencies. We can clearly see that the PeLEDs prepared with the Au NPs in the LELs possessed the largest deviation, indicating that the device was not very reproducible. Although the Au NPs we used here are amphiphilic, we suspect that the morphologies of the perovskite LELs were still affected, thereby limiting the device efficiencies. We foresee that proper surface modification of the Au NPs can help to reduce the negative impact on the morphology of the LELs [22].

We also blended the Au NPs into the first HTL, PTAA. As shown in Figure 3c, the addition of the Au NPs did not change the device characteristics greatly. Because the thickness of the VB-FNPD layer was about 40 nm, the plasmonic field induced by the NPs in the PTAA layer decayed exponentially and would not significantly influence the LELs of the PeLEDs. Therefore, the distance-dependent effects found in the PeLEDs also proved that the mechanism of the device enhancement was relevant to the plasmonic field of the nanostructures. Table 1 further summarizes the characteristics of the PeLEDs in this work.

**Figure 4:**
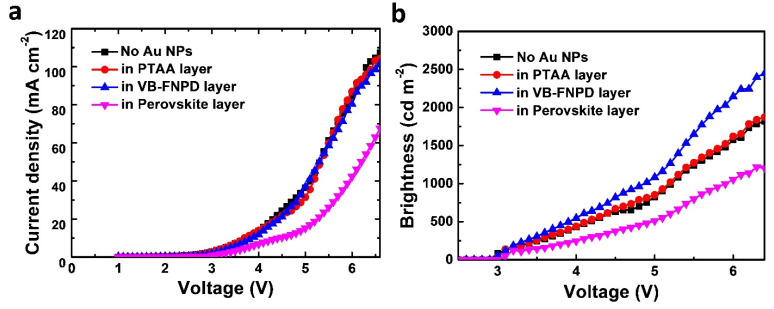
(**a**) Current density-voltage and (**b**) brightness-voltage characteristics of the PeLEDs containing Au NPs at different device locations. The concentration of the NPs was 5.0 × 10^−3^ mg mL^−1^.

Figure 4a displays the current density–voltage (*J*–*V*) and characteristics of the PeLEDs containing Au NPs at different device locations. We could not observe significant changes in the *J*–*V* curves after the Au NPs were incorporated into the VB-FNPD or PTAA layers. The applied currents were almost unchanged for these devices. However, the applied voltages were increased after the NPs were blended into the perovskite LELs, which was probably due to charge trapping. The balance between the electrons and holes was probably affected. Therefore, the electrical properties of the device were actually also affected significantly when the Au NPs were incorporated into the perovskite layers. Figure 4b also shows the brightness–voltage curves for the PeLEDs. The maximal brightness was improved from about 1750 to 2600 cd m^−2^ after the NPs were blended into the VB-FNPD layer. Meanwhile, the value was only increased slightly when the NPs were placed in the PTAA layer, confirming the influence was very limited. On the other hand, the brightness was decreased if the Au NPs were incorporated into the perovskite layer.

The log-log graph for the curves displayed in Figure 4a is re-plotted in Supplementary Materials Figure S1 for further analysis. At higher current regions, the curves could be described by *J* ∝ *V*^m+1^, according to the trapped space charge-limited current model [23]. The resulting m values of the devices were all very close to 4, indicating deep traps existed in the devices. For example, as indicated in Figure S1, the m value of the PeLED containing the NPs in the VB-FNPD layer was equal to 3.76. Moreover, we further investigated the differential resistance (*R*_diff_ = d*U*/d*I*) of the PeLEDs [24]. The results of the shunt resistance (*R*_sh_ = *R*_diff_(V = 0 V)) and the series resistance (*R*_s_ = *R*_diff_(V = 6 V)) for each condition are listed in Table S1. From the resistances, we can see that the *R*_s_ values were almost unchanged after the Au NPs were incorporated into the PTAA or VB-FNPD layer. The *R*_s_ decreased slightly after the Au NPs were blended in the perovskite layer. Similarly, the *R*_sh_ were very close to the PeLED prepared without Au NPs, confirming the electrical properties were not significantly changed after the use of the Au NPs. On the other hand, the *R*_sh_ increased after the Au NPs were blended into the perovskite layer. The morphologies of the perovskite LELs were possibly affected.

**Figure 5:**
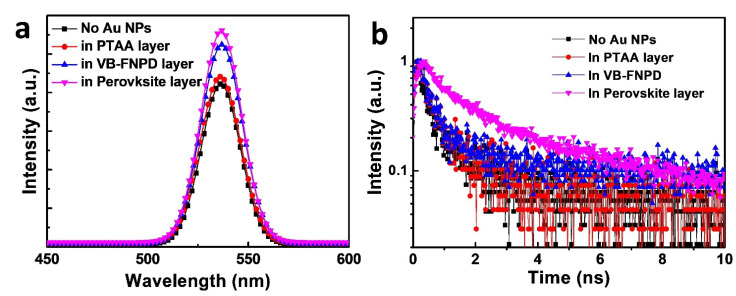
(**a**) The PL of the PeLEDs prepared with Au NPs at various locations in the devices. (**b**) time-reserved PL (TRPL) decay curves of the PeLEDs prepared under different conditions. The samples were prepared according to the device structure shown in Figure 1a, but no metal electrodes were deposited.

Figure 5a displays the steady-state PL spectra of the PeLEDs prepared with and without Au NPs. When the Au NPs were incorporated in the PTAA layer, we can see that PL intensity was only slightly increased [17,25,26]. The result supported the previous conclusion that the device efficiency was hardly affected because the NPs were far away from the light-emitting centers, i.e., the perovskite layer. On the other hand, if we blended the Au NPs in the VB-FNPD layer, PL was apparently enhanced, which was also consistent with the previous results, confirming their plasmonic effects [17,25]. More interestingly, when the Au NPs were incorporated into the perovskite layers, the device exhibited the highest intensity, suggesting the strongest plasmonic effects among the various configurations. Therefore, the PL results indicated that the direct blending of the Au NPs into the perovskite layer exhibited the most pronounced LSPR effects. In other words, the reason for the low efficiencies of the PeLED when using Au NPs in the LEL should be due to inferior electrical properties, such as poor charge transport or charge imbalance.

Figure 5b shows the corresponding time-resolved PL (TRPL) decay profiles for the PeLEDs prepared under various conditions. The curves could be fitted well using a biexponential decay equation [27,28]. The fast decay (τ_1_) could be assigned to the surface recombination, while the slower decay (τ_2_) represents radiative recombination [27]. Table 1 also summarizes the resulting fitting lifetimes for various types of PeLEDs. We can see clearly that the lifetimes became longer if the Au NPs were incorporated in the PeLEDs. The lifetimes slightly increased if the Au NPs were blended in the PTAA layer, due to the less effective consequences of the Au NPs. They further became longer when the Au NPs were incorporated into the VB-FNPD layer. The values of τ_1_ and τ_2_ could eventually be increased to 1.12 ± 0.01 and 7.75 ± 0.10 nsec, respectively, after the NPs were blended in the perovskite LELs. Although the details about the underlying mechanism are still under investigation, we suspect that the NPs could somehow reduce the level of surface and nonradiative recombination. The trend of TRPL results is consistent with that of steady-state PL measurements, confirming the distance-dependence of the position effects from the Au NPs.

Note that the phenomenon of the longer lifetime upon the use of NPs is different from the cases in polymer-based solar cells, where the lifetime becomes shorter [29]. The plasmonic effects provide an additional channel for exciton recombination, thereby reducing the lifetimes. For the PeLEDs in this work, on the other hand, two possible mechanisms could be probably responsible for the longer lifetimes of the perovskite films doped with Au NPs. The first is passivation of defects and/or reduced nonradiative recombination, as is described above. This could explain the enhanced PL intensities and longer lifetimes while the NPs were incorporated into the VB-FNPD and perovskite layers. However, because the PTAA layer did not make contact with the perovskite LEL directly, the slightly higher PL intensity and longer lifetime indicate some other factors could be also involved. Previously, Snaith et al. suggested that the addition of metal NPs in PSCs induces photon recycling (PR), which improves the device efficiencies of PSCs [30]. PR means re-absorption of radiative recombining charge pairs to generate an excitation, which can boost the external PLQY [31]; the lifetime may also become longer due to the possible enhanced PR. Therefore, the effect of the addition of NPs into the PeLEDs can probably also enhance PR in the PeLEDs, thereby improving the device efficiencies. In other words, the influence of PR could be more pronounced than opening additional channels for exciton recombination, thereby increasing the PL lifetimes for the devices prepared with NPs. However, further investigation is still required to validate this assumption.

In addition to plasmonic effects, some other mechanisms might be also responsible for the device improvements after the NPs are placed in the devices, such as the balance between electrons and holes [32,33]. However, as shown in Figure 4a, the *J–V* characterizations were not affected too much after the NPs were incorporated in the VB-FNPD layer, suggesting the change in electrical properties was not significant. Therefore, we deduced that the improved balance between electrons and holes should be not the main mechanism for device improvements. 

## 4. Conclusions

We have synthesized amphiphilic Au NPs for improving the device efficiencies of PeLEDs. Because the NPs can be dissolved in many different solvents, including water and DMF, they can be readily blended into different layers of the solution-processed PeLEDs; the special processing property allows us to study the position effects induced by the Au nanostructures. The PL studies indicate that the strongest plasmonic effects occur when the NPs are directly embedded in the perovskite LELs. In contrast, the optical properties are only affected slightly when they are blended into the PTAA layer, which is not directly in contact with the perovskite layer. After the Au NPs are blended into the second HTL, VB-FNPD layer, significant effects can be also observed; the distance-dependent phenomena reflect the near-field nature of the plasmonic field induced by the Au NPs. On the other hand, the PeLEDs prepared with Au NPs in the VB-FNPD HTL exhibit the highest luminance efficiency. As compared with the standard device, the addition of Au NPs in the VB-FNPD layer can improve the device efficiency by 42%. Unfortunately, directly blending the NPs in the perovskite layer might affect the electrical properties, resulting in inferior device performance. Further, the efficiency of PeLEDs is almost not affected after the NPs are used in the PTAA layers. Finally, from the PL studies, we deduce that the Au NPs probably can aid in passivating the defects, decreasing the level of nonradiative recombination and/or improving photon recycling. We anticipate that the results reported herein can help to understand the enhancing mechanism of plasmonic PeLEDs and also to lead to even better efficiencies in the near future.

## Figures and Tables

**Table 1:** Device and film properties of the PeLEDs as prepared in this work.

Device	Efficiency (cd A^−1^)	τ_1_ (nsec)	τ_2_ (nsec)
Standard (No Au NPs)	3.0 ± 0.4	0.34 ± 0.02	1.26 ± 0.15
in PTAA	3.0 ± 0.5	0.37 ± 0.03	2.01 ± 0.29
in VB-FNPD	4.2 ± 0.6	0.97 ± 0.07	5.49 ± 0.20
in perovskite layer	3.2 ± 0.9	1.12 ± 0.01	7.75 ± 0.10

## Data Availability

Data is contained within the article.

## Supplementary Materials

The following are available online at https://www.mdpi.com/article/10.3390/nano11040993/s1, Figure S1: The log–log plot of the J–V curves shown in Figure 4. The PeLEDs containing Au NPs at different device locations. The concentration of the NPs was 5.0 × 10^−3^ mg mL^−1^, Table S1: Differential resistances of the PeLEDs in this study.
