# Supplementary material for: Position Effects of Metal Nanoparticles on the Performance of Perovskite Light-Emitting Diodes
Source: Nanomaterials (Basel). 2021 Apr 13;11(4):993. doi: 10.3390/nano11040993 (PMC8068810; doi:10.3390/nano11040993)
Supplement: Supplementary file 1 [file nanomaterials-11-00993-s001.pdf]

Supplementary Information

Position Effects of Metal Nanoparticles on the Performance of Perovskite Light-Emitting Diodes

Chen-Min Yang [1] and Fang-Chung Chen [1,2,3,*]

[1] Department of Photonics, College of Electrical and Computer Engineering, National Chiao Tung University, Hsinchu 30010, Taiwan; dennis51025@gmail.com
[2] Center for Emergent Functional Matter Science, National Chiao Tung University, Hsinchu 30010, Taiwan
[3] Department of Photonics, College of Electrical and Computer Engineering, National Yang Ming Chiao Tung University, Hsinchu 30010, Taiwan
* Correspondence: fcchen@mail.nctu.edu.tw or fcchendop@nycu.nctu.edu.tw

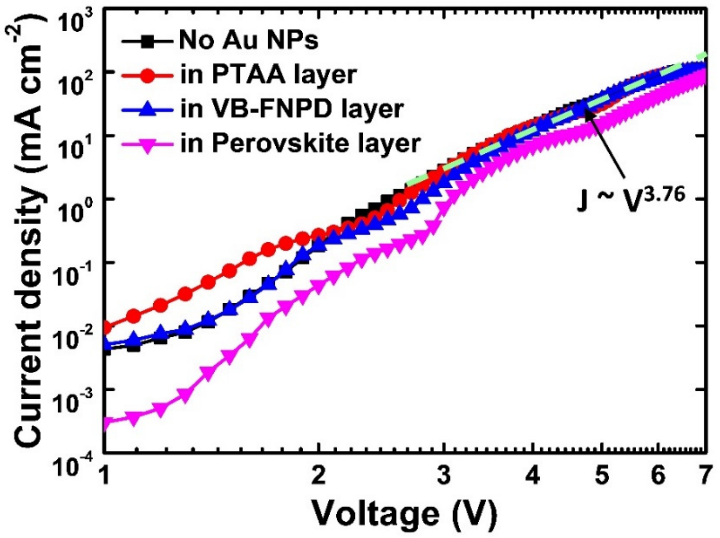

Figure S1. The log–log plot of the J-V curves shown in Figure 4. The PeLEDs containing Au NPs at different device locations. The concentration of the NPs was 5.0×10^{-3} mg mL^{-1}.

Table S1. Differential resistances of the PeLEDs in this study.

Device	Rs (Ω)	Rsh (Ω)
No Au NPs	6.41×10^2	7.84×10^4
in PTAA	5.53×10^2	4.67×10^4
in VB-FNPD	6.89×10^2	7.14×10^4
in Perovskite	4.02×10^2	2.31×10^5
